# Biological evaluation of solutions from bioglass, bioglass modified with cobalt, and calcium hydroxide

**DOI:** 10.1590/1807-3107bor-2025.vol39.114

**Published:** 2025-11-07

**Authors:** João Rafael AMADEU, Isabela Joane Prado SILVA, Juliana GOTO, Alexandre Henrique dos REIS-PRADO, Karina Sampaio CAIAFFA, Murilo Camuri CROVACE, Luciano Tavares Angelo CINTRA, Alberto Carlos Botazzo DELBEM, Cristiane DUQUE, Francine BENETTI

**Affiliations:** (a) Universidade Estadual Paulista – Unesp, School of Dentristry of Araçatuba, Department of Restorative Dentistry, Araçatuba, SP, Brazil.; (b) Universidade Federal de Minas Gerais – UFMG, School of Dentistry, Department of Restorative Dentistry, Belo Horizonte, MG, Brazil.; (c) Universidade Federal de São Carlos – UFSCar, Materials Engineering, Vitreous Materials Laboratory, São Carlos, SP, Brazil.; (d) Universidade Estadual Paulista – Unesp, School of Dentristry of Araçatuba, Department,of Pediatric Dentistry and Public Health, Araçatuba, SP, Brazil.

**Keywords:** Biocompatible Materials, Calcium Hydroxide, Cell Survival

## Abstract

This study evaluated the cytotoxicity, biocompatibility, and bioactivity potential of bioglass solutions (F18 and F18 with cobalt; F18-Co) compared to Ca(OH)_2_ solution, to determine their suitability for use in vital pulp therapy. F18 bioglass was prepared, with a part being doped with cobalt. The solutions were prepared at a 1:10 powder-to-water ratio. L929 fibroblasts viability was assessed (MTT assay; 24 and 48-h). Tubes containing fibrin sponges embedded with either the solutions or saline (control) were prepared (16 tubes/group) and immediately implanted into 16 rats (4 tubes/rat). At 7 and 30 days, the euthanized rat specimens were analyzed for inflammation and bioactivity. The ANOVA with Tukey’s test, or Kruskal-Wallis with Dunn’s test was performed (p < 0.05). Undiluted, 1:2, and 1:4 diluted solutions reduced cell viability at 24-h (p < 0.05). The 1:8 and 1:16 dilutions of F18 and F18-Co exhibited cell viability similar to that of the control (p > 0.05), whereas Ca(OH)_2_ was cytotoxic (p < 0.05). At 48-h, F18 dilutions (undiluted, 1:2, and 1:4) exhibited similar results to the control (p > 0.05). F18-Co at 1:8 and 1:16 dilutions increased cell viability compared to Ca(OH)_2_ (p < 0.05) and were similar to the control (p > 0.05). On day 7, moderate-to-severe inflammation (p > 0.05) and thick fibrous capsule were observed. On day 30, mild inflammation was observed in the control and F18-Co groups, moderate inflammation in F18 (p < 0.05), and mild inflammation in Ca(OH)_2_ (p > 0.05). The fibrous capsule was thin. None of the materials exhibited positive structure in von Kossa and polarized light analysis. The F18 and F18-Co solutions are cyto- and biocompatible; however, no bioactivity was observed.

## Introduction

Root canal treatment is considered the standard of care for decayed teeth with irreversible pulpitis, yielding highly favorable outcomes.^1^ However, pulpotomy plays a particularly important role in young permanent teeth by preserving the pulp tissue within the root canals and enabling complete root formation.^2^ The European Society of Endodontology has recently recommended vital pulp therapy for teeth with deep caries lesions and asymptomatic pulp exposures.^3^

Following coronal pulp amputation, various agents can be applied to treat the remaining pulp tissue.^4^ While considerable attention has been given to the medications and repair materials that come into direct contact with the pulp tissue, few studies have investigated the irrigating agents used during this procedure.^5,6^ Calcium hydroxide is a popular agent for direct pulp contact, primarily due to its ability to release hydroxyl and calcium ions.^4^ The elevated pH resulting from hydroxyl ions release can activate tissue enzymes such as alkaline phosphatase,^7^ promoting mineralization and tissue repair. This enzymatic activation also facilitates the release of phosphate ions, which combine with calcium ions from the bloodstream to form a calcium phosphate sediment^7^ that serves as a matrix for subsequent tissue mineralization.^7^ Lime water, derived from calcium hydroxide, serve as an irrigation solution in vital pulp therapy, benefiting tissue repair and supporting tissue homeostasis through ions dissociated from calcium hydroxide.^8^

The bioactivity of a material, defined by its ability to induce mineralized tissue formation, plays a critical role in promoting pulp tissue repair and the formation of tertiary dentin.^9^ Other materials recognized for their bioactivity include bioglass, which demonstrates high osteoinductive and osteoconductive activity.^9^ Specific compositions of bioglass are designed to release calcium, sodium, silicon, and phosphate ions, which are metabolized by the body and contribute to biological effects such as angiogenesis and antimicrobial activity.^10^ Moreover, bioglass has been shown to regulate osteoblastic proliferation and differentiation,^9^ with similar effects anticipated in with odontoblasts due to the morphological and functional similarities between these cell types.^11^ These materials contain high concentrations of sodium, maintaining a neutral or slightly alkaline pH, leading to the formation of bioactive layers.^9^ Increasing evidence highlights the positive effects of bioactive glass on tissue vascularization.^10^

Bioglass powder, when mixed with distilled water, has demonstrated a remarkable ability to promote dentin remineralization^12^and reduce mineral loss while preserving the surface integrity of enamel during dental bleaching.^13^ Its antimicrobial effects have also been demonstrated against Enterococcus faecalis.^14^ A recent study indicated that bioglass-based pastes are biocompatible, induce osteogenesis, and exhibit antimicrobial activity comparable to calcium hydroxide paste.^14^However, the effects of experimental solutions derived from these materials on non-mineralized connective tissues have not yet been explored. Given that bioglasses release ions into the surrounding environment, solutions formed by mixing these biomaterial powders with distilled water might could potentially benefit pulp tissue during vital pulp therapy. This potential benefit warrants further evaluation, as understanding the biological properties of these materials is essential before their clinical application on dental pulp. The addition of cobalt ions to vitreous bioparticles has been shown to induce tissue hypoxia and stimulate angiogenesis.^15^

A new highly reactive bioglass formulation that does not crystallize during processing has been developed, namely F18.^16,17^ This material was designed specifically for soft tissue regeneration,^16,18^ with demonstrated properties such as fibroblasts proliferation and potential biodegradability.^18^ These characteristics highlight its potential role in pulp tissue repair. Therefore, we propose doping F18 bioglass with cobalt ions. Thus, this study evaluated the cytotoxicity, biocompatibility, and bioactivity of experimental irrigating solutions prepared from bioglass F18 and cobalt-doped F18 bioglass. An irrigating solution derived from calcium hydroxide was used for comparison. Null hypotheses were adopted, suggesting that there are no differences among the solutions regarding (a) cell viability, (b) biocompatibility, and (c) bioactivity potential.

## Methods

### *In vitro* study

#### Synthesis of bioglass particles

The melting of F18 bioglass^19^ was made in an electric furnace at 1,200ºC / 4 h using a platinum crucible. The reagents used for the material synthesis included sodium carbonate (Na_2_CO_3_), potassium carbonate (K_2_CO_3_), calcium carbonate (CaCO_3_), phosphorus pentoxide (P_2_O_5_), magnesium oxide (MgO), zinc oxide (ZnO), and silicon dioxide (SiO_2_), as previously described.^19^ The reagents were purchased from Sigma-Aldrich (Inc, St Louis, USA) and Santa Rosa LTDA (City, Brazil). Prior to melting, the reagents were homogenized in a jar mill (12 h). The glass was poured into water to form glass frit, which was subsequently dried in an oven at 100ºC for 24 h. The dried frit underwent preliminary grinding in a planetary ball mill (500 rpm) for variable times (5–30 min). Further milling and sieving steps were conducted to obtain particles smaller than 25 µm. A separate batch of F18 glass doped with cobalt oxide (CoO, 1wt.%)^17,20^ was prepared under the same experimental conditions, including melting and milling.

#### Preparation of solutions

Experimental solutions of F18, F18-Co, and calcium hydroxide (Ca(OH)_2_) were produced by mixing the powdered materials with distilled water at a 1:10 powder-to-water ratio by weight.

#### Cytotoxicity analysis

L929 cell line fibroblasts were cultured under standard cell culture conditions in Dulbecco’s Modified Eagle’s medium (DMEM) supplemented with 10% fetal bovine serum (FBS), penicillin, and streptomycin, at 37ºC, 100% humidity, 95% air, and 5% CO_2_. The cells were seeded into 96-well plates (10^4^ cells/well) and incubated for 24 h under standard conditions to allow for cell adhesion prior to the addition of the solutions. For the preparation of solutions, distilled water was replaced with DMEM. Subsequently, various dilutions of the solutions were prepared in separate falcon tubes using DMEM as the diluent to achieve the following concentrations: undiluted, 1:2, 1:4, 1:8, and 1:16. The extracts were applied to the cells, and cell viability was assessed by the 3-(4,5-dimethylthiazol-2-yl)-2,5-diphenyltetrazolium bromide (MTT) assay at 24 and 48 hours. For the MTT assay, the culture medium and solution dilutions were removed from each well, and 100 µL of MTT solution (0.5 mg/mL in DMEM without FBS, 1:10) was added to each well. The MTT solution was removed after 4 hours of incubation, and the resulting formazan crystals were dissolved in 100 μL of isopropyl alcohol. The plate was then placed on a rotary shaker in a darkroom at room temperature for 30 minutes. Absorbance was measured at 570 nm using an Elisa reader (Eon Microplate Spectrophotometer, Biotek, City, USA).

## *In vivo* study

### Animals

Sixteen 2-month-old male Wistar albino rats (250–280 g) were used. The sample size was established based on the findings of a previous study.^21^ The animals were kept in an environment with temperature between 22 and 24ºC, controlled light cycle (12 hours light and 12 hours dark) and received food and water *ad libitum*. The local institutional ethics committee approved the experimental protocol (protocol 00198-2020). All procedures were conducted in accordance with ARRIVE guidelines 2.0.^22^

### Preparation of the tubes

For the *in vivo* analysis, sixty-four polyethylene tubes (Abbot Lab. Ltda., São Paulo, Brazil) with 1.0 mm internal diameter, 1.6 mm external diameter and 10.0 mm long were used (ISO 10993-6: 2007). The tubes were filled with fibrin sponges^23^ soaked in the experimental solutions. Sixteen tubes were prepared for each solution: F18, F18-Co and Ca(OH)_2_), as described above. An additional sixteen tubes containing fibrin sponges soaked in 0.9% saline solution served as the control group.

### Surgical procedure

**Figure 1:**
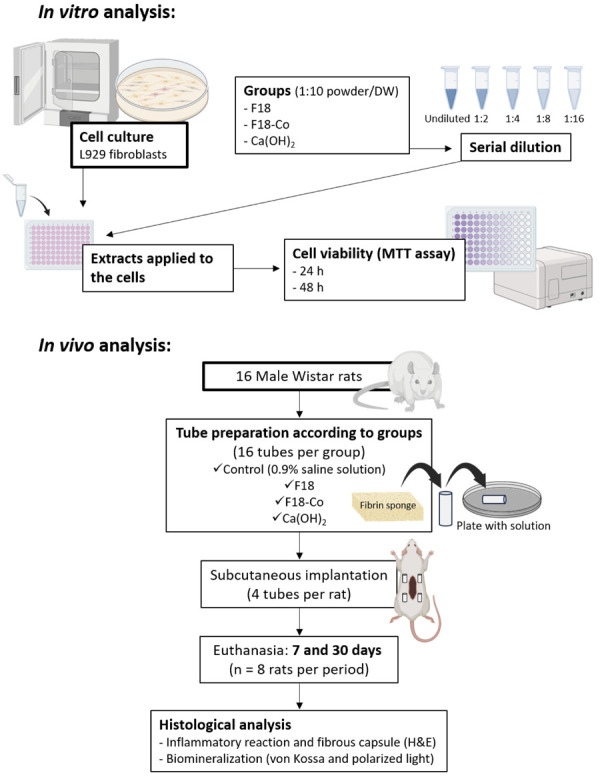
Study’s design flowchart.

The surgical procedure was performed as described in a previous study^21^ and is illustrated in Figure 1. The rats were anesthetized using intraperitoneal injection of ketamine (80 mg/kg, Francotar; Virbac do Brasil Ind e Com Ltda; Roseira, Brazil) and xylazine (10 mg/kg, Rompum; Bayer SA, São Paulo, Brazil). Their dorsal fur was shaved, and a 2.0-cm incision was made in a head-to-tail orientation with a #15 Bard-Parker blade (BD, Franklin Lakes, NJ, USA). The skin was reflected to create two pockets on the right side and two pockets on the left side of the incision. Thus, each animal received a total of four tubes: one from each experimental group (F18, F18-Co and Ca(OH)_2_) and one from the control group. The placement of the tubes in each pocket on the animals’ dorsum was randomized using a simple drawing method. Each pocket was assigned an identification number (1–4), and the drawing occurred during tube insertion process. Thus, the tissue was sutured with 4.0 non-resorbable silk thread (Ethicon, Johnson & Johnson, São Paulo, Brazil), and the final antisepsis was performed. The animals were monitored until they recovered from anesthesia. For postoperative analgesia, the rats received one subcutaneous application of 150 mg/Kg of dipyrone (Neo Química; São Paulo, Brazil). The rats were monitored daily using the Rat Grimace Scale, a partially automated method for quantifying pain in laboratory rats via facial expressions. If any signs of distress or suffering were detected, the animal would have been euthanized; however, no such interventions were required in this study. After completing the experimental procedures, groups were formed according to the material used, and outlined in Table 1.

**Table 1:** Distribution of experimental groups for 7 and 30 days

Groups	Solution inside the tube	n
Control	0.9% Saline solution	8
Ca(OH)_2_	Ca(OH)_2_ solution (1:10 powder for distilled water)	8
F18	F18 solution (1:10 powder for distilled water)	8
F18-Co	F18-Co solution (1:10 powder for distilled water)	8

### Histological analyses

At 7 and 30 days, the rats were euthanized (n = 8 rats per period, yielding 8 tubes from each group per time point) using an overdose of anesthetic (150 mg/kg, sodium thiopental, Thipentax, Cristália - Produtos Químicos Farmacêuticos LTDA, Itapira, Brazil). No animals were excluded from the study. The tubes, along with the surrounding tissues, were removed and fixed in a solution of 4% buffered formaldehyde for 24 h, at pH 7.0. The specimens were processed, embedded in paraffin, and sectioned serially at 5 μm for hematoxylin and eosin (HE) staining, while 10 μm histological sections were stained using the von Kossa (VK) technique or kept unstained for polarized light (PL) examination.^21^

Regarding the inflammation scores, fibrous capsule classification, and bioactivity analysis, histological sections were examined by a single experienced and calibrated operator in a blinded manner using light microscopy (Optiphot-2, Nikon, Japan). The tissue inflammation in contact with the materials at the open end of the tubes were scored based on previous study^21^: a) (no or few inflammatory cells and no reaction); b) (< 25 cells and mild reaction); c) (25–125 cells and moderate reaction); and d) (≥ 125 cells and severe reaction). The most central histological sections of the tube were used for score evaluation, with one section per specimen. For this analysis, the number of inflammatory cells in each group was quantified in an area of 1.2×0.6 mm (400 × magnification) at the center of the tube opening.^21^ Images were first captured, and then the inflammatory cells were marked using a computer program to perform the count (Leica QWin V3, Leica Microsystems, Wetzlar, Germany). The fibrous capsule was classified as thin (> 150 µm) or thick (≥ 150 µm).^21^ Additionally, VK-positive structures or birefringent structures under PL were recorded as either present or absent.^21^

## Statistical Analyses

Statistical analysis was performed by using Statistical Package for SigmaPlot (version 12.0, Systat Software Co., city, country) software program. The analyses compared the different groups across each period and, for cytotoxicity, within each concentration. Additionally, for cytotoxicity, the data from the control group were considered as 100%. The data obtained from the cytotoxicity analysis underwent a normality test and then the appropriate statistical test. Thus, after normality testing, one-way ANOVA with Tukey’s post hoc test was used for data analysis at 24 hours, while Kruskal-Wallis with Dunn’s post hoc test was applied for data analysis at 48 hours. The data obtained from the histological analysis were also subjected to the Kruskal-Wallis and Dunn tests. For all tests, statistical significance was set at α = 0.05.

## Results

### *In vitro* study - Cytotoxicity analysis

**Figure 2:**
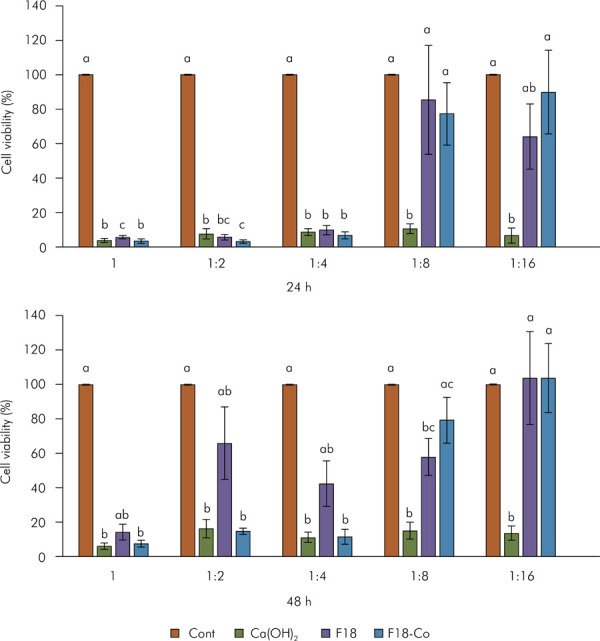
L929 fibroblasts’ viability determined by an MTT assay. After normality testing, one-way ANOVA with Tukey’s post hoc test was used for data analysis at 24-h, while Kruskal-Wallis with Dunn’s post hoc test was applied for data analysis at 48-h (p < 0,05). The same letters indicate no statistical differences among the groups at the same period and in the same dilution.

The data on cell viability are presented in Figure 2. A decrease in cell viability was observed for the undiluted materials solutions, as well as the 1:2 and 1:4 dilutions at 24 h, when compared to the control (p < 0.05). The other dilutions (1:8 and 1:16) of F18 and F18-Co solutions had lower cell viability than control, but these differences were not statistically significant (p > 0.05). In contrast, the 1:8 and 1:16 dilutions of Ca(OH)_2_ solutions showed significantly reduced cell viability compared to other groups (p < 0.05), except for the 1:16 dilution of the F18 solution, which did not differ in this period (p > 0.05).

At 48 h, the undiluted, 1:2, and 1:4 dilutions of Ca(OH)_2_ and F18-Co solutions decreased cell viability compared to the control (p< 0.05), whereas the F18 solutions (undiluted, and 1:2 and 1:4 dilutions) were similar to the control (p > 0.05). On the other hand, other dilutions of F18-Co (1:8 and 1:16) increased cell viability compared to the corresponding dilutions of the Ca(OH)_2_ solution (p < 0.05), being similar to the control (p > 0.05). The 1:8 dilution of F18 differed from to control (p < 0.05), while the 1:16 dilution was similar to the control (p > 0.05).

### *In vivo* study – Histological analyses

**Figure 3:**
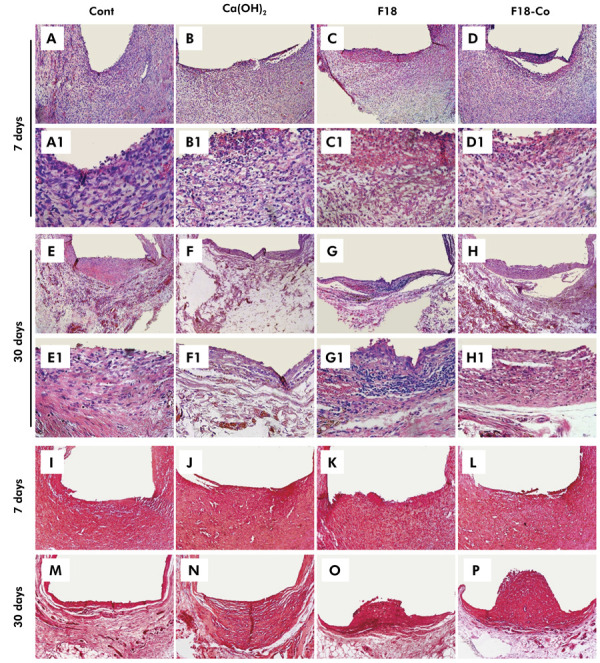
Representative images of the subcutaneous tissue reactions for inflammatory infiltrate and bioactivity by von Kossa. (a,a1 - h,h1) Inflammatory response images. At 7 days: (a,a1) control, (c,c1) F18, and (d,d1) F18-Co groups, with the presence of moderate inflammatory cell infiltration and a thick fibrous capsule, while (b,b1) Ca(OH)2 has severe inflammation. At 30 days: (e,e1) control and (h,h1) F18-Co groups with absence or mild inflammatory infiltration, (f,f1) Ca(OH)2 group with mild inflammation, and (g,g1) F18 groups with mild to moderate inflammation, and thin fibrous capsule. (i – p) Von Kossa staining images. (i – l) At 7 days and (m - p) at 30 days with absence of positive structures for von Kossa in all groups. [Hematoxylin-eosin staining, a-h - 100×, a1-h1 - 400×; von Kossa staining, i-p - 100×]

Representative images of the tissue responses are shown in Figure 3, and the histological analysis is provided in Table 2. At 7 days, a moderate infiltration of inflammatory cells was observed in the F18-Co group, while some specimens in the control and F18 groups showed moderate inflammatory infiltrate, and others exhibited severe inflammatory infiltrate. The Ca(OH)_2_ group showed severe infiltration of inflammatory cells in most specimens during this period. In some specimens across all material groups, an area of superficial necrosis was observed in the tissue region in contact with the material. However, no significant differences were observed among the groups (p > 0.05), and all specimens exhibited a thick fibrous capsule.

**Table 2:** Inflammatory score and thickness of fibrous capsule according to group, at 7 and 30 days.

Time (days)	p-value	Groups	Scores				Median*	Capsule		n
			1	2	3	4		Thick	Thin	
7	0.385	Control	0	0	4	4	3,5^a^	8	0	8
		Ca(OH)_2_	0	0	3	5	4^a^	8	0	
		F18	0	0	4	4	3,5^a^	8	0	
		F18-Co	0	1	5	2	3^a^	8	0	
30	0.005	Control	6	2	0	0	1^a^	0	8	8
		Ca(OH)_2_	2	5	1	0	2^ab^	1	7	
		F18	1	3	4	0	2,5^b^	1	7	
		F18-Co	6	2	0	0	1^a^	0	8	

*Same letters indicate no statistical difference concerning the inflammatory response among the groups in each analysis period (p > 0.05).

At 30 days, most specimens from the control and F18-Co groups showed either an absence of or few inflammatory cells (p >0.05), whereas the F18 group primarily exhibited mild to moderate inflammation (p <0.05). The Ca(OH)_2_ showed mild inflammation in most specimens, with no significant differences compared to the other groups (p >0.05). The fibrous capsules were thin in most specimens across all groups at this time point.

**Figure 4:**
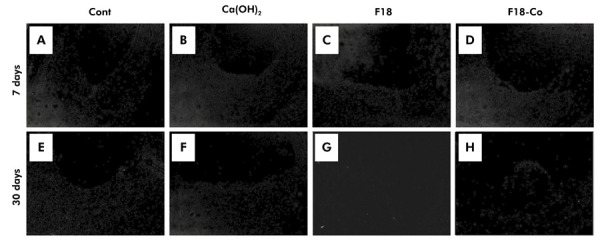
Representative images of the polarized light analysis. At 7 days, (a) control, (b) Ca(OH)2, (c) F18, and (d) F18-Co groups, and at 30 days, (e) control, (f) Ca(OH)2, (g) F18, and (h) F18-Co groups, with absence of birefringent structures in all groups. [Polarized light analysis, a-h - 100×]

Regarding the bioactivity analyses, all experimental materials exhibited no positivity for VK staining (Figure 3) and no birefringent structures to PL (Figure 4) at both 7 and 30 days.

## Discussion

This study analyzed the cell viability, biocompatibility, and bioactivity potential of solutions from bioglass materials (F18 and F18 doped with cobalt), compared with a solution from Ca(OH)_2_, as potential candidates for vital pulp therapy. In vitro analysis involved the use of both pure extracts and various extract dilutions, following ISO standards,^24^ and guided by previous research.^21^ This approach was chosen because, following material application in tissues, leachable substances are gradually cleared by extracellular fluids, leading to a gradual decrease in local concentrations, as reflected by the range of dilutions used. Furthermore, employing undiluted extracts may result in rapid cell death, which differs from *in vivo* biocompatibility studies.^11,21^ In the present *in vivo* analysis, the undiluted solution, which was the primary focus of our research, was used. The results indicated that more diluted solutions of F18 and F18-Co (1:8 and 1:16) maintained cell viability at 24 hours, as did most solutions of F18 and F18-Co at 48 hours, in contrast to the Ca(OH)_2_ solution. Thus, the null hypothesis regarding (a) cell viability of the solutions was rejected. However, all solutions were biocompatible, mainly F18-Co, and none solution showed bioactivity *in vivo*. Therefore, the null hypotheses regarding (b) biocompatibility and (c) bioactivity potential among the tested solutions were accepted.

Considerable efforts are being made to explore various materials suitable for permanent contact with pulp tissue in vital pulp therapy.^2-4^ However, the irrigants used during these procedures have not been extensively studied. The most commonly studied irrigating solutions are sodium hypochlorite and chlorhexidine.^25,26^Nevertheless, the results for both are controversial. Some studies suggest that they may delay pulp repair,^27,28^ and sodium hypochlorite has been shown to cause severe cytotoxic effects even at low concentrations.^29^

Ca(OH)_2_ is known to induce pulp tissue repair.^14,30^ It has been observed that Ca(OH)_2_ paste allows the formation of dentin in pulpotomy treatments^30^ and yields satisfactory clinical results.^31^ Additionally, it exhibits excellent antimicrobial action.^14^ On the other hand, an irrigating solution prepared by mixing saline solution with Ca(OH)_2_ powder demonstrates low antimicrobial effect but provides excellent hemostatic activity due to the activation of coagulation factors by the release of calcium ions.^8^

Bioactive glass is gaining increasing popularity in dentistry.^12^ When vitreous materials come into contact with aqueous solutions, a time-dependent kinetic modification occurs on their surface,^9,32^ resulting in the formation of a bond between the material and the surrounding tissues. For instance, the formation of a layer of hydroxycarbonate apatite (HCA) on the surface of bioglass – resulting from the dissolution of the glass in the aqueous medium – facilitates its attachment to dental or bone tissues.^12,32,33^ HCA interacts with collagen fibrils, promoting integrating with the tissues.^9^ These leaching reactions of bioactive glasses have been well described previously.^9^

When bioglass is placed in an aqueous solution, it releases alkaline elements, leading to an increase in pH.^9,32^ Concomitantly, Si-O-Si bonds break through the action of hydroxyl ions (OH), releasing silica into the solution in the form of silicic acid [Si(OH)_4_].^9,32,34^ During the precipitation process, calcium and phosphate ions released from the bioglass form a layer rich in calcium phosphate (CaP),^32^ initially amorphous, which later transforms into HCA.

Previously, F18 bioglass membranes showed an enhancement of osteoblastic cell metabolic activity,^33^ with increased in cell viability observed in fibroblast lineage cells. However, in this study, the pure F18 solution demonstrated the most rapid increase in cell viability compared to the other evaluated materials over a 48 h-hour period. When evaluated in membrane form, a rapid response in cell growth was also observed,^33^ attributed to its fast dissolution into particles, releasing ions such as Ca, Na, Si, and P into the medium.^34^ Moreover, F18 induced in vitro proliferation of osteoblast and fibroblast cells.^16,18,34,35^ These data are consistent with the results observed in the present study.

The rapid ionic dissociation observed in our in vitro analysis may have been beneficial to fibroblasts. This ionic release is beneficial for tissues requiring repair, as it activates mesenchymal stem cells involved in the healing process.^33,36^ Similarly, the F18-Co solution also showed satisfactory results compared to the Ca(OH)_2_ solution. Previous studies have revealed that doping materials with transition metals, such as cobalt, can improve their anticancer, antioxidant, and antimicrobial activities.^37,38^ Additionally, cobalt doping has been found to improved the biological, magnetic, and optical properties of materials used in biomedicine.^39^ When incorporated into bioglass, cobalt has been observed to stimulate angiogenesis.^15^ Therefore, bioglass solutions could be alternative irrigants that act directly on pulp tissue and promote its repair.

Bioglass was first tested for biocompatibility in 1981 using discs implanted in the subcutaneous tissue of rats.^9^ The material exhibited positive interaction with connective tissue, making it suitable for clinical use.^9^ Bioglass was also evaluated as a scaffold in subcutaneous rat tissue, resulting in reduced chronic inflammation over a 45-day implantation period, along with the formation of a fibrous capsule.^35^ These findings are consistent with the results presented in this study.

Bioactive materials have shown the capacity to enhance hard tissue formation when interacting with mesenchymal stem cells from dental pulp.^36^ As a result, several investigations using histological analyses have been performed to identify biomaterials that can induce osteogenic differentiation of stem cells upon contact with dental pulp.^21,36^ The successful restoration of the damaged dentine-pulp complex, characterized by the deposition of a hard tissue barrier between the remaining vital pulp and the restorative material, serves as a key clinical indicator of treatment success.^30^

Previously, F18 fibers used for bone regeneration demonstrated enhanced biocompatibility and bioactivity in rat subcutaneous tissue and cell culture.^18^ In addition, a previous *in vivo* study showed the degradability of F18 following the implantation of its fibers into the subcutaneous tissue of rats, with notable formation of HCA on the material’s surface.^18^ This suggests that, in addition to its bioactivity, F18 has significant potential for regenerating connective tissues, supporting tissue repair processes.

In this investigation, the bioactivity of the biomaterial solutions was analyzed using VK staining and PL. These methods can reveal positive structures if the material, upon contact with tissue, exhibits bioactivity.^21^ This bioactivity is associated with the reaction of calcium ions from the evaluated materials with carbonic dioxide from the tissue, leading to the localized production of calcite crystals in the surrounding area^21^. These biological events may be associated, for example, with the production of a tertiary dentin barrier after pulp capping or pulpotomy treatments.

While a previous article reported the formation of HCA on the surface of F18 fibers,^18^ this study found no positive or birefringent particles at the fibrous capsule site in contact with the F18 and F18-Co solutions. Similarly, birefringent structures to PL and positive for VK staining were absent in the Ca(OH)_2_ solution. These findings differ from the existing literature, where the formation of a hard tissue barrier has been reported after pulp capping with the Ca(OH)_2_ in human teeth.^27,28,30^ Various variables may have influenced these findings, including differences in the experimental models used for bioactivity analysis, such as direct capping of human pulps versus subcutaneous tissue response in rats. More importantly, the solution form used in this study may have impacted our results on bioactivity analysis due to a low concentration of the materials present in the solutions.

Although the beneficial effects of cobalt doping were mentioned in this study, this is the first study to evaluate an F18 solution with cobalt, and the results found here were promising. For instance, it is essential to assess whether these solutions might influence the repair materials that come into contact with pulp tissue during vital pulp therapy. This evaluation is particularly crucial for mineral trioxide aggregate, which is considered the gold standard repair material for these treatments, as well as for other bioceramic materials used for similar purposes.

The biocompatibility data were positive, showing absent to mild inflammation in the F18-Co group after 30 days of implantation, which did not occur with the other materials. However, the solutions of all materials did not showed bioactivity in the connective tissue of rats. The limitations of this study should be acknowledged. Although both in vitro and *in vivo* studies provide preliminary insights, they are crucial for the analysis of new materials. Our in vitro assay exhibits the limitation wherein only the 24 and 48-h periods were used to analyze the cytotoxicity of the extracts. However, the *in vivo* model permits analysis after 7 d and 30 d of tissue contact with materials, which is important, as a material is considered biocompatible when tissue inflammation in contact with it is reduced over time.^40^

Another limitation of the study is the use of rat subcutaneous tissue for testing the solutions, rather than pulp tissue. Odontoblasts in the pulp tissue, when exposed to bioglass irrigating solutions, might be more susceptible to show bioactivity. However, the chosen model did not permit this analysis. Evaluations in rat subcutaneous tissue require fewer animals and help guide the selection of the optimal materials for more specific tissue testing. Thus, these findings are preliminary, and further studies are needed to assess the effects of these solutions in direct contact with pulp tissue and to determine whether cobalt enhances angiogenesis. Future research should also investigate the antimicrobial properties of these solutions, their reactions upon contact with pulp tissue, and the potential use of F18 doped with cobalt as repair material for direct pulp capping or pulpotomy models, particularly concerning hard tissue formation.

## Conclusion

Diluted experimental solutions of F18 bioglass and F18Co were cytocompatible, and all tested solutions were biocompatible; however, no *in vivo* bioactivity was observed in any of the evaluated solutions.

## Data Availability

The datasets generated during and/or analyzed during the current study are available from the corresponding author on reasonable request.
